# Spatial and Vertical Stratification of Groundwater Microbial Communities Reveals Proteobacterial Dominance and Redox-Driven Ecological Transitions

**DOI:** 10.3390/microorganisms14010232

**Published:** 2026-01-19

**Authors:** Rahaf S. Aljuaid, Sahar A. Alshareef, Basma T. Jamal, Ftoon H. Dhafeer, Alaa A. Alnahari, Ruba A. Ashy

**Affiliations:** 1 Department of Biology, College of Science & Arts at Khulis, University of Jeddah, Jeddah 21493, Saudi Arabia; raljuaid0022.stu@uj.edu.sa (R.S.A.); salshareef2@uj.edu.sa (S.A.A.); 2 King Fahd Medical Research Center, King Abdulaziz University, Jeddah 22252, Saudi Arabia; btjamal@kau.edu.sa; 3 Department of Biological Sciences, College of Science, University of Jeddah, Jeddah 21493, Saudi Arabia; 2315316@uj.edu.sa (F.H.D.); aamalnahari@uj.edu.sa (A.A.A.)

**Keywords:** *16S rRNA* gene amplicon sequencing, groundwater microbiome, Pseudomonadota (Proteobacteria), redox gradients, aquifer hydrogeochemistry

## Abstract

Groundwater microbial communities exhibit pronounced vertical and spatial structuring driven by physicochemical gradients. Here, we investigated microbial assemblages across surface and subsurface layers of three groundwater wells distributed along a 1.26 km transect in the Wadi Awja aquifer system (Jeddah, Saudi Arabia) using high-throughput *16S rRNA* gene amplicon sequencing. Across all samples, Pseudomonadota (Proteobacteria) dominated community composition, accounting for ~50–65% of surface assemblages and increasing to ~90% in deeper strata, indicating strong vertical selection. This depth-associated enrichment coincided with reduced community evenness and the prevalence of metabolically versatile, facultatively anaerobic taxa. Although Actinomycetota, Bacteroidota, and Planctomycetota contributed substantially to overall diversity, their relative abundances declined with depth, reinforcing the dominance of Proteobacteria under suboxic conditions. Notably, members of Enterobacteriaceae, particularly Escherichia spp., were consistently enriched in deeper layers, coinciding with simplified community structures. Collectively, these results demonstrate that groundwater microbial communities undergo sharp redox-associated ecological transitions over short spatial scales, emphasizing the role of localized hydrogeochemical heterogeneity in shaping subsurface microbial assemblages.

## 1. Introduction

Groundwater ecosystems are inhabited by complex microbial communities that play fundamental roles in biogeochemical cycling, including organic carbon turnover, nutrient transformations, and contaminant attenuation [1,2,3]. These subsurface microbiomes also act as sensitive sentinels of aquifer health, with shifts in community structure frequently mirroring variations in redox potential, oxygen gradients, and anthropogenic pressures such as agricultural runoff and industrial pollution [4,5,6]. Understanding these microbial assemblages is therefore crucial for predicting ecosystem responses to environmental stress and for maintaining sustainable groundwater quality in the face of increasing exploitation.

Vertical stratification is a defining feature of groundwater ecosystems. Typically, surface and shallow zones harbor higher microbial richness and evenness, dominated by aerobic and chemoorganotrophic taxa such as *Pseudomonas*, *Limnohabitans*, and members of *Verrucomicrobiota*, reflecting oxic and nutrient-enriched conditions [7,8]. In contrast, deeper layers tend to exhibit reduced diversity, favoring facultative and obligate anaerobes such as *Enterobacteriaceae*, *Shewanella*, and *Desulfovibrio*, which thrive under oxygen-limited and electron-acceptor-dependent environments [1,2,3,4,5,6,7,8,9]. This vertical organization reflects strong redox and geochemical zonation, where metabolic versatility and adaptation to energy limitation determine microbial survival and community turnover [10,11].

Despite extensive research in temperate and alluvial aquifers, the microbial ecology of groundwater in arid and coastal regions remains underexplored. These systems are highly susceptible to salinization, seawater intrusion, and over-extraction, processes that alter ionic composition, redox conditions, and the distribution of organic substrates, thereby reshaping microbial community structure [12,13,14]. Previous studies have suggested that in such systems, microbial distribution often aligns more closely with hydrogeochemical gradients—such as salinity, dissolved oxygen, and redox potential—than with spatial distance per se [15,16,17]. Consequently, evaluating microbial composition along short transects can reveal the relative influence of physicochemical heterogeneity versus spatial separation in shaping subsurface biodiversity.

The Red Sea coastal plain represents a hydrogeologically sensitive aquifer system characterized by high ambient temperatures, variable salinity, limited recharge, and pronounced redox gradients, collectively creating challenging conditions for groundwater microbial communities [18,19]. In arid and coastal aquifer systems worldwide, groundwater microbiomes are frequently dominated by metabolically versatile taxa—particularly members of the phylum Proteobacteria—whose distribution is governed more strongly by hydrochemical parameters, such as salinity, dissolved oxygen, and redox state, than by spatial distance alone. Despite increasing interest in subsurface microbial ecology, baseline microbiological information from shallow coastal aquifers in western Saudi Arabia remains scarce, and fine-scale spatial and vertical structuring of groundwater microbial communities over short transects is poorly documented. This knowledge gap limits ecological interpretation and constrains effective assessment of groundwater vulnerability in rapidly developing coastal regions.

By integrating high-throughput *16S rRNA* gene sequencing with hierarchical taxonomic profiling, this study aims to: (i) characterize microbial community structure across surface and depth layers; (ii) assess spatial differentiation among wells separated by short and long distances; and (iii) identify key taxa that may serve as bioindicators of aquifer redox dynamics and vulnerability. Through this approach, we provide novel insights into how physicochemical gradients and environmental constraints shape the vertical and spatial architecture of groundwater microbiomes in arid coastal ecosystems.

## 2. Materials and Methods

### 2.1. Study Area and Sampling

**Figure 1:**
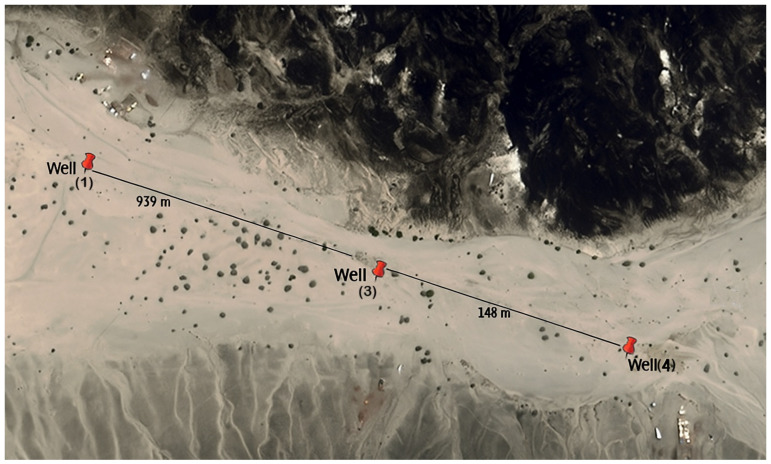
Geographic location of groundwater sampling wells (W1, W3, and W4) along a 1.26 km transect on the Red Sea coastal plain. Inter-well distances are indicated (W1–W3: 939 m; W3–W4: 148 m), illustrating the spatial arrangement of sampling sites used for groundwater microbial community analysis. Sampling was conducted during a single field campaign on 24 February 2025. Groundwater flow direction and hydraulic connectivity among wells were not directly measured and are therefore not inferred from this map. Scale bar: 400 m. Image © 2025 Airbus (Google Earth).

Groundwater samples were collected from three hand-dug wells (W1, W3, and W4) located along a 1.26 km transect on the Red Sea coastal plain near Jeddah, Saudi Arabia (Figure 1). The wells were positioned at 22°3.595′ N, 39°14.360′ E (W1), 22°3.116′ N, 39°14.579′ E (W3), and 22°3.039′ N, 39°14.595′ E (W4), with inter-well separations of approximately 939 m (W1–W3) and 148 m (W3–W4). Each well was sampled at two vertical strata: surface water (S; ~20 cm below the water surface) and a deeper interval (D; 1.5–2.5 m below the water surface, depending on well depth), with two independent replicates per stratum (S1–S2 and D1–D2), yielding a total of 12 groundwater samples (Figure 1; Table 1). Sampling was conducted during a single morning field campaign on 24 February 2025 (07:30–09:30) under ambient air temperatures of 19–29 °C. Samples were collected using pre-sterilized 1 L high-density polyethylene (HDPE) containers attached to nylon ropes with a cleaned weight. Surface samples were taken directly from the upper water layer, whereas depth samples were obtained by lowering a covered container equipped with a balloon seal, which was released upon reaching the target depth. Upon retrieval, water temperature was measured immediately using a calibrated thermometer, and samples were transferred aseptically into sterile 50 mL Falcon tubes and pre-labeled according to well and sampling depth (e.g., W1-S1, W3-D2). Samples were transported on ice in insulated coolers and processed in the laboratory within three hours of collection. Upon receipt, aliquots intended for *16S rRNA* gene amplicon sequencing were refrigerated and subsequently transported to King Abdullah University of Science and Technology (KAUST), while remaining aliquots were fixed by adding 2 mL of 25% glutaraldehyde per 50 mL sample for long-term preservation. Glutaraldehyde-fixed samples were stored at 4 °C in the dark as a preservation backup for potential downstream cell-based analyses (e.g., total cell enumeration or microscopy-based assessments). These preserved aliquots were not processed further in the present study and were not available for retrospective quantitative analyses at the time of manuscript preparation. Because site-specific hydraulic head measurements were not available, groundwater flow direction and hydraulic connectivity among wells were not directly quantified; therefore, spatial interpretations are based on well positions and inter-well distances (Figure 1) and are discussed in relation to localized physicochemical heterogeneity and community ordination patterns reported in the Results. Photographs of the wells and the sampling procedure are provided in Supplementary Figure S1.

### 2.2. DNA Extraction and Sequencing

All groundwater samples were transported on ice immediately after collection and arrived at the laboratory within approximately 3 h. Filtration for DNA extraction was performed on the same day of sampling upon arrival at King Abdullah University of Science and Technology (KAUST). Samples were stored at 4 °C for less than 24 h prior to filtration and DNA extraction. No samples were frozen prior to filtration. While transport and short-term storage may result in minor shifts in physicochemical conditions (e.g., oxygen exposure), all samples were handled identically, minimizing systematic bias across wells and depths. Genomic DNA was extracted from microbial pellets obtained by centrifuging 500 mL of groundwater from each sample at 10,000 × g for 10 min. The resulting pellets from Wells W1, W3, and W4—each comprising surface (S1, S2) and depth (D1, D2) replicates—were processed using the DNAbler Cell and Tissue Kit (Haven Scientific, Jeddah, Saudi Arabia) following the manufacturer’s protocol. DNA purity and concentration were assessed using a NanoDrop spectrophotometer (Thermo Fisher Scientific, Waltham, MA, USA). The V3–V4 hypervariable regions of the *16S rRNA* gene were amplified using the universal primers 341F (5′-CCTACGGGNGGCWGCAG-3′) and 806R (5′-GACTACHVGGGTATCTAATCC-3′). Amplicon libraries were prepared using the Illumina Nextera XT kit and sequenced on an Illumina NovaSeq 6000 platform (Illumina, Inc., San Diego, CA, USA) (paired end, 150 bp reads). This approach provided comprehensive coverage of the V3–V4 region and enabled high-resolution taxonomic profiling of microbial communities across surface and depth layers of Wells W1, W3, and W4. Absolute microbial abundance was not quantified in this study. Quantitative PCR was not feasible because no residual DNA extracts were available following amplicon library preparation.

### 2.3. Bioinformatics and Sequence Processing

Raw sequence data generated from the Illumina NovaSeq 6000 platform were analyzed using a standardized *16S rRNA* gene amplicon bioinformatics workflow. Initial quality assessment was conducted using FastQC (https://www.bioinformatics.babraham.ac.uk/projects/fastqc/, accessed on 15 October 2025) to evaluate base quality distribution, GC content, and sequence length profiles. Adapter and primer sequences were trimmed using Cutadapt, and low-quality bases were further removed with Trimmomatic (http://www.usadellab.org/cms/?page=trimmomatic, accessed on 10 December 2025), ensuring consistent read length and a minimum Phred quality score above 25. The filtered reads were imported into QIIME 2 (version 2023.2) (https://qiime2.org) for denoising, quality control, and amplicon sequence variant (ASV) inference using the DADA2 plugin (https://library.qiime2.org/plugins/q2-dada2, accessed on 15 December 2025). DADA2 performed quality filtering, error correction, merging of paired-end reads, and removal of chimeric sequences, yielding high-resolution ASVs with single-nucleotide accuracy. The resulting feature table was filtered to retain ASVs of appropriate V3–V4 fragment length using R (version 4.3.1). Taxonomic classification of representative ASVs was performed with a Naïve Bayes classifier trained on the SILVA 138 SSURef NR99 database (https://www.arb-silva.de), trimmed to the same primer set (341F/806R). Chloroplast- and mitochondria-affiliated sequences were removed prior to rarefaction and downstream analyses. Organelle-derived reads were absent from the final dataset (0.00% across all samples), indicating negligible host or phototrophic contamination and high data quality. The resulting ASV abundance table was normalized by sequencing depth and used for subsequent analyses of alpha and beta diversity, community composition, and taxonomic profiling across groundwater wells W1, W3, and W4.

### 2.4. Taxonomic Assignment and Phylogenetic Classification

Taxonomic annotation of high-quality amplicon sequence variants (ASVs) was performed using a Naïve Bayes classifier implemented in the QIIME 2 feature-classifier plugin (https://docs.qiime2.org), trained on the SILVA rRNA reference database (version 138) (https://www.arb-silva.de). The reference sequences were trimmed to match the amplified V3-V4 region defined by primers 341F and 806R to ensure consistent classification accuracy. Taxonomic assignments were generated across hierarchical levels from phylum to species, with confidence scores (bootstrap support ≥ 50%) used to validate lineage placement. Unclassified reads and singletons were excluded from downstream analyses. To infer phylogenetic relationships among ASVs, multiple-sequence alignment was conducted using MAFFT (version 7.526) (https://mafft.cbrc.jp/alignment/software/, accessed on 10 December 2025), and a midpoint-rooted phylogenetic tree was constructed using FastTree 2 (http://www.microbesonline.org/fasttree/, accessed on 12 December 2025). The resulting taxonomy table and phylogenetic tree were subsequently integrated into the QIIME 2 environment for diversity analyses and community composition visualization.

### 2.5. Alpha Diversity and Rarefaction Analyses

Alpha diversity was assessed to evaluate microbial richness, evenness, and phylogenetic diversity across groundwater wells W1, W3, and W4. Following quality control and ASV inference, the feature table was rarefied once to an even sequencing depth of 35,000 reads per sample, and all subsequent diversity and statistical analyses were conducted using this rarefied dataset to minimize bias arising from unequal sequencing depth. Alpha diversity indices, including Observed ASVs, Chao1, Shannon, Simpson, Gini–Simpson, and Faith’s Phylogenetic Diversity (PD), were calculated using the QIIME 2 diversity plugin (https://qiime2.org, version 2023.2). Rarefaction curves were generated using the qiime diversity alpha-rarefaction command (1000 iterations) to verify sequencing depth sufficiency and coverage of community diversity. Observed ASVs and Chao1 indices were used to estimate richness, whereas Shannon and Simpson indices described community evenness and dominance. Faith’s PD incorporated phylogenetic relationships inferred from a tree constructed using FastTree 2 (http://www.microbesonline.org/fasttree/, accessed on 12 December 2025). Statistical differences in alpha diversity among wells and between surface and depth samples were evaluated using nonparametric Kruskal–Wallis tests with a significance threshold of *p* < 0.05. Visualization of alpha diversity patterns was performed using QIIME 2 View and R (version 4.3.1) with the phyloseq and vegan packages.

### 2.6. Top of Form

#### Beta Diversity and Community Structure

Beta diversity analyses were performed within the QIIME 2 environment (version 2023.2; https://qiime2.org) to assess differences in microbial community composition among groundwater samples from Wells W1, W3, and W4. Pairwise dissimilarity matrices were computed using both Bray–Curtis (abundance-based) and weighted UniFrac (phylogeny-based) distance metrics. Prior to analysis, ASV tables were rarefied to an even depth of 35,000 sequences per sample to minimize sequencing bias. Principal Coordinates Analysis (PCoA) plots were generated using the qiime diversity pcoa and qiime emperor plot functions to visualize clustering patterns. Statistical differences among wells were tested using PERMANOVA (permutational multivariate analysis of variance, 999 permutations) via the qiime diversity beta-group-significance plugin (https://library.qiime2.org/plugins/q2-dada2, accessed on 15 December 2025). Visualization and confirmation analyses were additionally conducted in R (version 4.3.1) (https://www.r-project.org, accessed on 21 May 2025) using the phyloseq and vegan packages (https://joey711.github.io/phyloseq, accessed on 10 December 2025). Hierarchical clustering was performed using the UPGMA algorithm based on Bray–Curtis dissimilarity, and dendrogram branch lengths represent dissimilarity values.

### 2.7. Statistical Analyses

All statistical analyses were performed after rarefaction of the ASV table to a uniform sequencing depth to minimize bias associated with unequal sequencing effort. Alpha diversity metrics, including Observed ASVs, Chao1 richness, Shannon diversity, Simpson, Gini–Simpson, and Faith’s Phylogenetic Diversity (PD), were statistically compared among wells (W1, W3, and W4) and between sampling depths (surface vs. depth) using non-parametric Kruskal–Wallis tests, as data did not meet assumptions of normality. Statistical significance was assessed at *p* < 0.05. Beta diversity was evaluated using Bray–Curtis dissimilarity as well as weighted and unweighted UniFrac distance matrices. Differences in community composition associated with well identity and sampling depth were tested using permutational multivariate analysis of variance (PERMANOVA) with 999 permutations. Due to the limited number of biological replicates per depth (n = 2), replicate-level statistical comparisons were not performed. Instead, replicate consistency was assessed qualitatively by comparing taxonomic profiles, relative abundance patterns, and ordination clustering of paired samples (S1–S2 and D1–D2). Replicates within each well and depth exhibited concordant community structures, supporting downstream ecological interpretation.

To identify the taxa driving differences in microbial community composition among groundwater wells, a similarity percentage (SIMPER) analysis was performed at the genus level using Bray–Curtis dissimilarity. Amplicon sequence variant (ASV) tables were first collapsed to genus level and converted to relative abundances. SIMPER analyses were conducted for pairwise comparisons among wells (W1 vs. W3, W1 vs. W4, and W3 vs. W4) using the *vegan* package in R. The analysis quantified the average contribution of each genus to the overall Bray–Curtis dissimilarity between groups. Results are reported descriptively, highlighting the dominant genera cumulatively explaining the majority of between-well dissimilarity. No hypothesis testing was applied, as SIMPER is an exploratory method intended to identify taxa contributing most strongly to observed community differences.

## 3. Results

### 3.1. Sequencing Quality Control

High-throughput sequencing of the *16S rRNA* gene V3–V4 region generated paired-end reads for all groundwater samples collected from wells W1, W3, and W4. Raw read counts per sample ranged from 117,110 (W3-D2) to 195,601 (W1-S1), with a mean of approximately 153,000 reads per sample.

Following adapter and primer trimming using Cutadapt and quality filtering with DADA2, 70.8–87.1% of reads were retained across samples. The number of quality-controlled reads after filtering (QC remain) ranged from 84,066 (W3-D1) to 164,516 (W1-S1).

Denoising with DADA2 produced high-confidence forward and reverse reads that were subsequently merged into complete amplicon pairs. The number of merged reads per sample ranged from 65,264 to 148,902. Chimera removal reduced read counts by approximately 10–15%, resulting in non-chimeric read totals between 65,264 (W3-D1) and 141,485 (W1-D2).

After length filtering, final amplicon sequence variant (ASV) read counts ranged from 65,264 to 141,485 per sample. Retention efficiency, calculated as the proportion of ASV reads relative to raw input reads, ranged from 53.4% to 83.4%. The highest retention efficiency was observed in sample W1-D2 (83.39%), whereas samples W1-S2 and W3-S2 exhibited lower retention values (~53–54%). Detailed sequencing statistics for each preprocessing step are summarized in Supplementary Table S1.

### 3.2. Phylum-Level Composition

**Figure 2:**
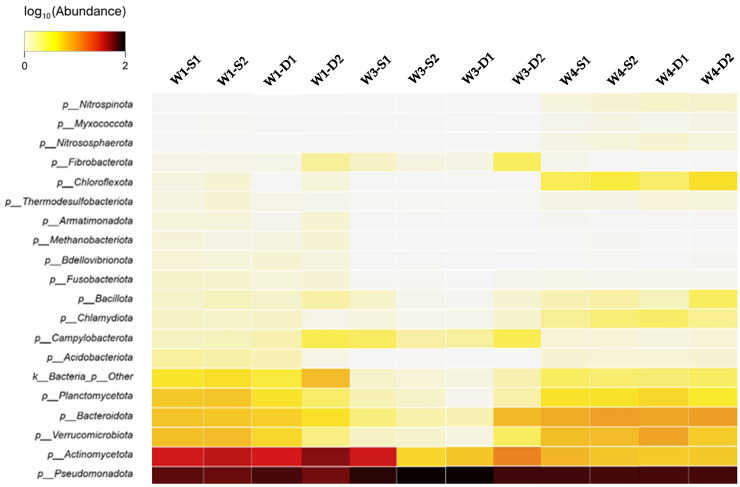
Heatmap showing the relative abundance of bacterial and archaeal phyla across individual groundwater samples from wells W1, W3, and W4. Columns represent individual samples collected from surface (S1–S2) and depth (D1–D2) intervals and were analyzed independently (no pooling). Samples are ordered by well identity and sampling depth to facilitate comparison of spatial and vertical patterns. Rows correspond to detected phyla and are ordered by hierarchical clustering based on relative abundance profiles. Color intensity reflects log_10_-transformed relative abundance, with lighter colors indicating lower proportions and darker colors indicating higher proportions.

A total of 32 bacterial and archaeal phyla were detected across groundwater samples collected from wells W1, W3, and W4 (Figure 2). Sequences affiliated with the domain Bacteria accounted for >99.5% of reads in all samples, whereas Archaea represented a minor fraction (<0.5%) and were primarily assigned to Methanobacteriota and Nitrososphaerota.

In Well W1, both surface samples (W1-S1, W1-S2) and depth samples (W1-D1, W1-D2) were dominated by Pseudomonadota and Actinomycetota. In surface samples, Pseudomonadota accounted for approximately 45–55%, while Actinomycetota represented 20–25% of sequences. Depth samples showed comparable phylum composition, with Pseudomonadota comprising 50–60% and Actinomycetota 20–23%. Additional phyla detected consistently in both surface and depth samples included Verrucomicrobiota (≈6–9%), Bacteroidota (≈5–8%), and Planctomycetota (≈4–6%). Other phyla, including Bacillota, Campylobacterota, and Acidobacteriota, each accounted for less than 1% of sequences. Archaeal Methanobacteriota were detected at low relative abundance (≈0.1–0.3%) across W1 samples.

In Well W3, phylum-level composition was strongly dominated by Pseudomonadota in both surface (W3-S1, W3-S2) and depth samples (W3-D1, W3-D2). Surface samples contained approximately 70–80% Pseudomonadota, while depth samples reached 85–90%. Actinomycetota represented 8–15% of surface samples and decreased in depth samples (5–10%). Bacteroidota occurred at low relative abundances (≈2–4%), whereas Verrucomicrobiota, Planctomycetota, and other phyla each contributed less than 2% across all W3 samples. Archaeal sequences were rare, accounting for <0.05% of reads in both surface and depth samples.

In Well W4, both surface (W4-S1, W4-S2) and depth samples (W4-D1, W4-D2) exhibited a more evenly distributed phylum-level profile compared with W3. In surface samples, Pseudomonadota accounted for approximately 55–65%, while Actinomycetota represented 6–8%, Bacteroidota 8–12%, Verrucomicrobiota 5–8%, and Planctomycetota 3–5%. Depth samples showed an increase in Pseudomonadota (65–75%) and corresponding decreases in Actinomycetota, Bacteroidota, and Planctomycetota. Chloroflexota were consistently detected in W4 samples at low to moderate relative abundances (≈2–4%). Archaeal Nitrososphaerota were most frequently detected in this well (≈0.1–0.3%), though remained a minor component overall.

Across all wells, replicate samples collected from the same depth interval (surface or depth) displayed similar phylum-level profiles. Differences in phylum composition were more pronounced among wells than between surface and depth samples within individual wells, while consistent depth-associated shifts—particularly increased relative abundance of Pseudomonadota in deeper samples—were observed across the dataset (Figure 2).

#### Vertical Stratification Across Groundwater Depths

Across all three wells, clear vertical stratification was observed between surface (S) and deeper (D) groundwater samples. Surface communities were characterized by higher taxonomic evenness and greater relative contributions from Actinomycetota, Bacteroidota, and Planctomycetota. In contrast, deeper strata exhibited pronounced enrichment of Pseudomonadota, reaching up to ~90% relative abundance in some samples, accompanied by reduced alpha diversity and increased dominance of Gammaproteobacteria. These depth-associated shifts were consistent across wells, indicating a coherent vertical ecological signal independent of spatial separation (Figure 2; Table S1).

### 3.3. Sequencing and Taxonomic Composition at Class Level

Taxonomic profiling at the class level identified more than 60 bacterial and archaeal classes across groundwater samples collected from wells W1, W3, and W4 (Supplementary Figure S2). Across all samples, the majority of sequences were assigned to classes within the phylum Pseudomonadota (Proteobacteria), with Gammaproteobacteria, Betaproteobacteria, and Alphaproteobacteria representing the dominant classes in both surface (S) and depth (D) samples.

In Well W1, surface samples (W1-S1, W1-S2) exhibited a relatively even class-level composition, dominated by Betaproteobacteria (≈25–29%), Gammaproteobacteria (≈12–16%), and Alphaproteobacteria (≈8–10%). Actinomycetes (≈15–18%), Planctomycetia (≈4–6%), Verrucomicrobiia (≈4–6%), Cytophagia (≈2–4%), and Flavobacteriia (≈2–4%) were consistently detected. Depth samples (W1-D1, W1-D2) showed a similar class-level composition, with a higher relative abundance of Gammaproteobacteria compared to surface samples, while Betaproteobacteria and Alphaproteobacteria remained present at comparable levels.

In Well W3, surface samples (W3-S1, W3-S2) were characterized by strong dominance of Gammaproteobacteria (≈60–70%), with Actinomycetes (≈9–12%), Betaproteobacteria (≈3–5%), and Alphaproteobacteria (≈2–4%) detected at lower relative abundances. Depth samples (W3-D1, W3-D2) exhibited the most pronounced Gammaproteobacteria dominance observed across all wells, reaching up to ≈90% of sequences, while other classes occurred at low relative abundances (<5%). Archaeal classes were detected only sporadically and at very low levels in both surface and depth samples from this well.

In Well W4, surface samples (W4-S1, W4-S2) displayed a broader class-level distribution, with Gammaproteobacteria (≈35–40%), Alphaproteobacteria (≈10–12%), Betaproteobacteria (≈9–11%), and Actinomycetes (≈5–7%) representing the dominant classes. Additional classes included Flavobacteriia (≈10–12%), Verrucomicrobiia (≈6–8%), Planctomycetia (≈2–3%), Phycisphaerae (≈1–2%), and Caldilineae (≈2–3%). Depth samples (W4-D1, W4-D2) showed a comparable class-level composition, with Gammaproteobacteria increasing in relative abundance relative to surface samples, while Alphaproteobacteria and Betaproteobacteria remained consistently present. Archaeal Nitrososphaeria were detected at low relative abundance (≈0.1–0.3%), primarily in depth samples.

Across all wells, replicate samples collected from the same depth interval (S or D) displayed comparable class-level profiles. Differences between surface and depth samples were primarily reflected in shifts in the relative abundance of Gammaproteobacteria, whereas overall class-level composition remained consistent within individual wells (Supplementary Figure S2).

### 3.4. Genus-Level Composition

At the genus level, groundwater microbial communities exhibited clear compositional differences among wells W1, W3, and W4 (Supplementary Figure S3). Genus-level assignments are reported descriptively and should be interpreted with caution, as they are based on partial *16S rRNA* gene sequences and may not fully resolve closely related taxa.

In Well W1, both surface samples (W1-S1, W1-S2) and depth samples (W1-D1, W1-D2) displayed a relatively diverse genus-level composition with multiple taxa present at moderate relative abundances. Actinomycetota-affiliated genera collectively accounted for approximately 14–17% of sequences across S and D samples, while *Pseudomonas* represented approximately 8–12%. Additional recurrent genera detected in both surface and depth samples included *Polynucleobacter* (≈5–8%), members of the *Burkholderia/Comamonadaceae* group (≈5–7%), and *Aestuariivirga* (≈2–4%). Several other genera, including *Cellulophaga*, *Sandaracinomonas*, *Fontisphaera*, *Oleiharenicola*, and *Haloferula*, were consistently detected at lower relative abundances (generally 1–3%) across replicates.

In Well W3, genus-level composition differed markedly between wells but remained consistent between surface (W3-S1, W3-S2) and depth samples (W3-D1, W3-D2). *Escherichia* (≈30–40%) and *Rheinheimera* (≈20–30%) dominated both surface and depth samples, together accounting for more than half of the total sequences in this well. Actinomycetota-associated genera were detected at lower relative abundances (≈7–10%), while members of *Burkholderiales* represented approximately 2–4%. Most remaining genera occurred at low relative abundances (<2%) across all W3 samples.

In Well W4, surface samples (W4-S1, W4-S2) and depth samples (W4-D1, W4-D2) exhibited a more evenly distributed genus-level profile compared to W3. The most abundant genera across both depth intervals included *Rheinheimera* (≈28–35%), *Cellulophaga* (≈7–11%), *Haloferula* (≈5–7%), and Actinomycetota-associated genera (≈5–7%). Additional genera such as *Limnohabitans* (≈6–8%), *Aquiluna* (≈3–5%), *Aestuariivirga*, *Fontisphaera*, and *Pseudomonas* were consistently detected at lower relative abundances across surface and depth samples.

Across all wells, replicate samples collected from the same depth interval (surface or depth) exhibited similar genus-level profiles. Differences in genus-level composition were primarily observed among wells rather than between surface and depth samples within the same well, consistent with patterns observed at higher taxonomic ranks (Supplementary Figure S3).

### 3.5. Alpha Diversity and Rarefaction Analyses

Alpha diversity metrics were evaluated separately for surface (S) and depth (D) samples within each groundwater well, and results are presented at the individual-sample level without pooling (Table 2). Rarefaction curves based on observed ASVs, Chao1 richness, Shannon diversity, Faith’s Phylogenetic Diversity (PD), and Gini–Simpson indices approached saturation for all samples, indicating that sequencing depth was sufficient to capture the majority of microbial diversity.

In Well W1, surface samples (W1-S1, W1-S2) exhibited high alpha diversity, with Shannon indices of 5.31–5.38 and Faith’s PD values of 86.8–89.3. Depth samples (W1-D1, W1-D2) showed comparable diversity, with Shannon values ranging from 4.90 to 5.03 and Faith’s PD from 67.8 to 82.2.

In Well W3, both surface and depth samples displayed consistently lower alpha diversity relative to the other wells. Surface samples showed Shannon indices of 3.21–3.48 and Faith’s PD values of 36.0–38.6, while depth samples ranged from 3.59 to 4.30 in Shannon diversity and 20.2–30.2 in Faith’s PD.

In Well W4, surface samples (W4-S1, W4-S2) exhibited high diversity, with Shannon indices of 5.11–5.48 and Faith’s PD values of 73.5–88.6. Depth samples (W4-D1, W4-D2) maintained similarly elevated diversity, with Shannon values of 4.61–4.85 and Faith’s PD of 67.7–70.0.

Across all wells, replicate samples collected from the same depth interval (S1–S2 or D1–D2) showed comparable alpha diversity values. Differences between surface and depth samples were therefore assessed descriptively, as the limited number of biological replicates (n = 2 per depth) does not permit robust statistical testing. Overall, alpha diversity patterns were consistent within wells while differing among wells, as summarized in Table 2.

### 3.6. ASVs Richness Across Groundwater Samples

Amplicon sequence variant (ASV) richness was evaluated descriptively for surface (S) and depth (D) samples within each groundwater well based on rarefaction analysis (Supplementary Figure S4). Within each well, replicate samples from the same depth interval (S1/S2 and D1/D2) showed comparable rarefaction trajectories, indicating consistent sequencing coverage and reproducible richness estimates. In Well W1, surface replicates (W1-S1 and W1-S2) exhibited higher ASV richness (480–506 ASVs) than depth replicates (W1-D1 and W1-D2; 340–450 ASVs). The rarefaction curves for S1 and S2 closely overlapped, as did those for D1 and D2, supporting their treatment as within-depth replicates. In Well W3, ASV richness was consistently lower than in the other wells across both depth intervals. Surface replicates (W3-S1 and W3-S2) yielded 181–203 ASVs, whereas depth replicates (W3-D1 and W3-D2) showed reduced richness (112–130 ASVs). Replicate curves within each depth interval were closely aligned, indicating internally consistent richness patterns. In Well W4, surface samples (W4-S1 and W4-S2) exhibited the highest ASV richness among all wells (457–548 ASVs), while depth samples (W4-D1 and W4-D2) showed moderately lower but comparable richness values (402–403 ASVs). As in the other wells, replicate samples within each depth interval displayed similar rarefaction behavior. Across all wells, surface samples tended to exhibit higher ASV richness than corresponding depth samples; however, these observations are reported descriptively only. Given the limited number of replicates per depth (n = 2), no statistical testing was performed, and differences are interpreted qualitatively based on replicate consistency and relative trends.

### 3.7. Beta Diversity and Microbial Community Structure

**Figure 3:**
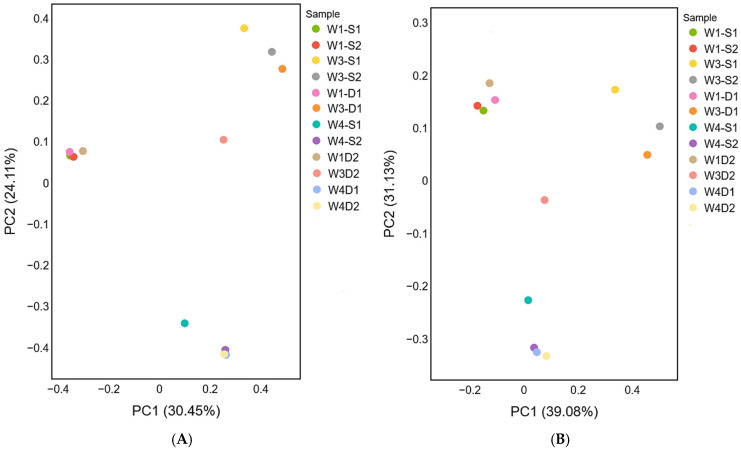
Principal Coordinates Analysis (PCoA) plots illustrating beta diversity of groundwater microbial communities from wells W1, W3, and W4. (**A**) Bray–Curtis dissimilarity–based PCoA showing differences in amplicon sequence variant (ASV) relative abundance among surface (S1, S2) and depth (D1, D2) samples. (**B**) Unweighted UniFrac distance–based PCoA depicting phylogenetic dissimilarity among microbial communities. Each point represents an individual sample, colored by well. Axes are labeled with the percentage of variance explained.

Beta diversity analyses were conducted using Bray–Curtis dissimilarity and unweighted UniFrac distance metrics to evaluate compositional and phylogenetic differences among groundwater microbial communities from wells W1, W3, and W4. Principal coordinates analysis (PCoA) based on Bray–Curtis dissimilarity (Figure 3A) showed that the first two axes explained 30.45% (PC1) and 24.11% (PC2) of the total variance, respectively. Samples formed distinct clusters corresponding to individual wells, with replicate samples from each well grouping closely together.

PCoA based on unweighted UniFrac distances (Figure 3B) explained 39.08% of the variation along PC1 and 31.13% along PC2. Similar to the Bray–Curtis analysis, samples clustered by well, with limited overlap between wells. Replicate samples within each well exhibited consistent positioning in ordination space across both distance metrics.

Overall, beta diversity analyses demonstrated well-specific clustering patterns that were reproducible across taxonomic (Bray–Curtis) and phylogenetic (unweighted UniFrac) distance measures.

### 3.8. SIMPER Analysis Identifying Taxa Driving Community Dissimilarity

To identify the genera contributing most to the compositional differences observed in the beta-diversity analysis, SIMPER analysis based on Bray–Curtis dissimilarity was performed. The analysis revealed that a limited number of genera accounted for the majority of the dissimilarity between groundwater wells. In the comparison between W1 and W3, *Rheinheimera* and *Escherichia* were the strongest contributors, jointly explaining more than 34% of the total dissimilarity, followed by *Polynucleobacter* and *Cellulophaga*. Together, the top eight genera accounted for approximately 80% of the Bray–Curtis dissimilarity between the two wells (Table S2). These results indicate that the observed community separation is primarily driven by shifts in the relative abundance of a small subset of indicator genera rather than uniform changes across the entire microbial community.

### 3.9. UPGMA—Bray–Curtis Clustering

**Figure 4:**
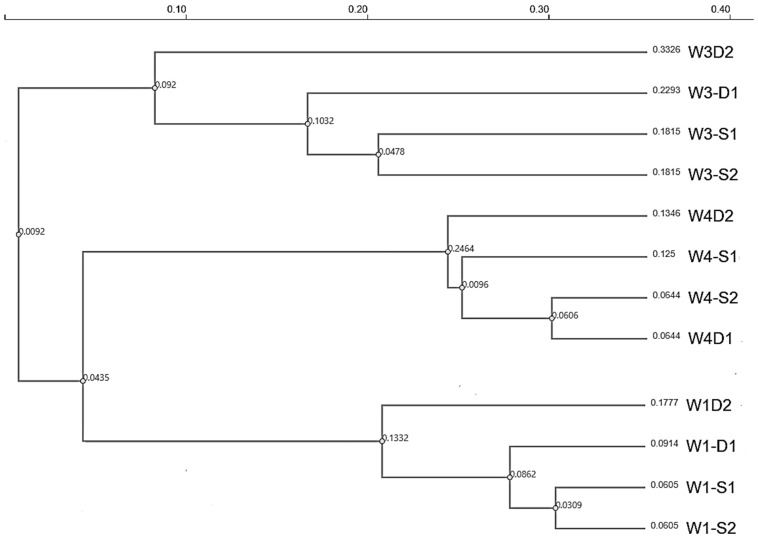
UPGMA dendrogram based on Bray–Curtis dissimilarity illustrating the hierarchical clustering of groundwater microbial communities from wells W1, W3, and W4 using genus-level relative abundance data. Numbers shown at internal nodes indicate the Bray–Curtis dissimilarity values at which clusters merge, while numbers displayed at the tips of branches represent the cumulative dissimilarity (branch length) of each sample relative to the root of the dendrogram.

Hierarchical clustering based on Bray–Curtis dissimilarity revealed a clear well-specific structuring of microbial communities (Figure 4). Samples from each well clustered together, indicating that well identity was the primary driver of community composition. In particular, all Well 3 (W3) samples (W3-S1, W3-S2, W3-D1, and W3-D2) formed a distinct and distant cluster, reflecting the highest overall dissimilarity relative to the other wells. Within this W3 cluster, S and D samples grouped closely, demonstrating internal consistency rather than dispersion.

Similarly, samples from Wells 1 (W1) and 4 (W4) formed separate, well-defined clusters, with S and D samples clustering together within each well. Minor branch-length differences were observed between S and D samples within the same well; however, these differences were markedly smaller than those separating different wells. This pattern indicates that while sample type (S vs. D) contributes to within-well variation, it does not override the dominant effect of well-specific environmental conditions on microbial community structure.

## 4. Discussion

### 4.1. Redox-Driven Structuring of Groundwater Microbial Communities

The observed dominance of Proteobacteria across all wells, together with their marked enrichment in deeper strata, underscores redox-driven ecological filtering as a primary determinant of groundwater microbial community assembly. Rather than reflecting simple depth effects, these patterns indicate selective advantages for metabolically flexible taxa under suboxic and dynamically fluctuating geochemical conditions. Consistent with this framework, Proteobacterial enrichment with depth coincided with reduced community evenness and the predominance of facultatively anaerobic lineages capable of exploiting alternative electron acceptors as oxygen availability declined.

Across karst, alluvial, and coastal aquifer systems, Proteobacteria are consistently reported as dominant constituents of groundwater microbiomes, with their distribution governed more strongly by hydrochemical parameters—such as dissolved oxygen, salinity or electrical conductivity, dissolved organic matter quality, and redox indicators—than by spatial distance alone. Large multi-well studies further demonstrate that microbial community composition is closely linked to localized hydrogeochemistry, such that environmental gradients can structure community similarity even within a single aquifer system [20,21]. Decade-scale syntheses of carbonate aquifers further indicate that groundwater microbiomes can retain functional resilience despite pronounced compositional turnover under changing recharge and hydrochemical conditions, reinforcing the role of localized heterogeneity in shaping distinct microbial signatures over short spatial scales [22].

### 4.2. Mechanistic Basis of Proteobacterial Enrichment Along Depth Gradients

Mechanistically, the pronounced enrichment of Proteobacteria with depth aligns with redox zonation as a first-order driver of groundwater microbial assembly. Progressive oxygen depletion along vertical gradients results in a sequential shift in terminal electron acceptors (O_2_ → NO_3_^−^ → Mn(IV)/Fe(III) → SO_4_^2−^ → CO_2_), favoring taxa capable of flexible respiration and efficient carbon utilization under energy-limited conditions.

Proteobacteria are particularly successful along this continuum due to their metabolic plasticity, enabling transitions between aerobic respiration, denitrification, metal reduction, and fermentation as electron-acceptor availability fluctuates. This functional versatility provides a robust explanation for their persistence and dominance under suboxic to mildly reducing conditions, indicating redox-driven ecological transitions rather than simple depth effects [1,2,9,23].

### 4.3. Well-Specific Differentiation and Fine Scale Hydrogeochemical Heterogeneity

Within this redox-driven framework, the contrasting microbial profiles of the studied wells are ecologically coherent. Wells W1 and W4 exhibited higher richness, evenness, and broader phylogenetic representation, consistent with more stable or transitional oxygen and resource regimes [22,23]. In contrast, Well W3 showed pronounced diversity loss and dominance of Gammaproteobacteria and Enterobacteriaceae, a pattern widely associated with deterministic selection under redox instability, elevated organic inputs, or localized hydrogeochemical stress [4,5,6,7,8,9,10,11].

In coastal plain aquifers such as the Red Sea setting investigated here, salinity gradients and salt–freshwater mixing can rapidly reorganize microbial communities and associated functions, often overriding spatial proximity and promoting sharp ecological differentiation over short distances [24,25]. These processes provide a parsimonious explanation for the distinct microbial signature of Well W3 relative to Wells W1 and W4, reinforcing the role of fine-scale hydrogeochemical heterogeneity as a dominant driver of groundwater microbial structure [16,17,18,19,20,21,22].

Comparable patterns of well-specific clustering and depth-associated compositional shifts have been widely reported across karst, alluvial, and coastal aquifers, where microbial community similarity within individual wells often exceeds similarity between neighboring wells over short spatial scales. This reflects strong control by localized hydrogeochemical conditions rather than spatial distance alone [5,17,26]. In this context, the consistent clustering of replicate surface (S) and depth (D) samples within each well observed here is ecologically meaningful and indicates that vertical variation operates as a secondary filter superimposed on dominant well-specific environmental constraints.

Beyond community-level patterns, the functional traits of the dominant genera provide mechanistic insight into the observed beta-diversity structure. Genera identified by SIMPER analysis as major contributors to between-well dissimilarity—such as *Rheinheimera*, *Escherichia*, *Polynucleobacter*, *Cellulophaga*, and *Limnohabitans*—are known to differ markedly in metabolic strategies and redox tolerance. *Rheinheimera* and *Escherichia* are facultatively anaerobic and frequently associated with suboxic or redox-fluctuating aquatic environments, whereas *Polynucleobacter* and *Limnohabitans* are typically linked to oxic freshwater systems with higher organic matter turnover. Similarly, *Cellulophaga* is involved in the degradation of complex organic polymers, indicating localized differences in carbon availability and substrate quality. The dominance of these taxa in specific wells therefore suggests that shifts in redox regime, organic carbon sources, and electron-acceptor availability are primary drivers of community differentiation rather than stochastic variation.

Collectively, these findings align with an emerging consensus that groundwater microbial communities are structured by deterministic environmental filtering, where functional traits of a limited number of dominant taxa disproportionately shape beta-diversity patterns. The integration of ordination, hierarchical clustering, and SIMPER analysis in this study therefore provides a coherent ecological framework linking community-level dissimilarity to the functional ecology of indicator genera, consistent with observations from diverse aquifer systems worldwide [9,27,28].

### 4.4. Ecological Interpretation of Enterobacteriaceae Enrichment

The enrichment of Enterobacteriaceae in Well 3, particularly sequences assigned to *Escherichia*, coincided with reduced alpha diversity and strong dominance patterns [4,5,6,7,8,9,10,11]. However, species-level interpretation must be treated with caution. It is well established that short-read *16S rRNA* gene amplicon sequencing, including the V3–V4 region, cannot reliably resolve closely related taxa within the *Escherichia–Shigella* clade, nor distinguish *Escherichia fergusonii* from *E. coli* or *Shigella* species with high confidence [29]. Accordingly, the detected sequences are best interpreted as belonging to the *Escherichia/Shigella* complex rather than definitive species-level assignments.

Importantly, enrichment of Enterobacteriaceae was observed consistently across all four W3 samples (surface and depth replicates), arguing against a single-sample contamination event. Nevertheless, the possibility of episodic fecal input or surface-derived organic loading—common in shallow, unprotected wells—cannot be fully excluded [15,16]. In the absence of complementary fecal markers, quantitative PCR, or metagenomic resolution, Enterobacteriaceae enrichment should therefore be interpreted as an ecological signal associated with redox instability or localized nutrient enrichment rather than definitive evidence of contamination [17,30,31].

### 4.5. Archaeal Rare Biosphere and Functional Implications

Although archaeal lineages such as Methanobacteriota and Nitrososphaerota were detected at low relative abundance, their presence aligns with growing evidence that rare archaeal taxa are persistent and ecologically meaningful components of groundwater microbiomes [9,10,11]. Previous studies have demonstrated that ammonia-oxidizing and methanogenic archaea can contribute disproportionately to nitrogen and carbon cycling under suboxic to anoxic conditions, despite limited representation in *16S rRNA* gene datasets [32,33,34]. Accordingly, the detection of these archaeal groups in the present study likely reflects underlying redox stratification and niche specialization rather than incidental occurrence, reinforcing the functional relevance of low-abundance taxa in subsurface biogeochemical processes. The strong clustering by well, particularly the distinct separation of W3, suggests that site-specific hydrogeochemical conditions exert a stronger influence on microbial community composition than sample type, which primarily contributes to fine-scale variation within wells.

Rarefaction was applied to standardize sequencing depth across samples and minimize bias in beta-diversity comparisons; however, this approach may reduce sensitivity to extremely low-abundance taxa. Despite this limitation, rare archaeal groups were consistently detected across samples, suggesting that major ecological patterns were retained and that rarefaction did not eliminate the core rare biosphere signal.

Future investigations employing deeper sequencing, non-rarefied compositional methods, or shotgun metagenomics will be required to fully resolve rare microbial populations and their functional roles under fluctuating redox conditions.

### 4.6. Study Limitations and Future Directions

Despite providing novel insights into fine-scale spatial and vertical structuring of groundwater microbial communities along the Red Sea coastal plain, the present study has several limitations that should be considered when interpreting the results.

First, because samples were not filtered in situ, short-term alterations in physicochemical conditions—particularly oxygen availability—during transport cannot be fully excluded. However, all samples were handled using identical protocols and processed within a narrow time window, supporting the robustness of relative comparisons of spatial and vertical community structure.

Second, microbial community analyses were based on relative abundances derived from *16S rRNA* gene amplicon sequencing, and absolute microbial abundance was not quantified. Because no residual DNA extracts or preserved water aliquots remained available after sequencing preparation, quantitative PCR-based enumeration or cell-based staining approaches could not be applied. Consequently, observed taxonomic enrichments should be interpreted as compositional shifts rather than confirmed increases in absolute cell numbers.

Third, groundwater flow direction and hydraulic connectivity among wells were not directly measured due to the absence of site-specific hydraulic head data. As a result, interpretations of spatial differentiation necessarily reflect localized physicochemical heterogeneity rather than inferred advective transport or dispersal pathways.

Fourth, the limited number of biological replicates per depth (n = 2) precluded robust statistical testing. Replicate consistency was therefore evaluated qualitatively based on concordant taxonomic profiles, relative abundance patterns, and ordination clustering, which consistently supported the reported spatial and vertical trends.

Fifth, microbial biomass was concentrated by centrifugation rather than membrane filtration, which may have resulted in incomplete recovery of very small or low-density cells, including ultramicrobacteria. Although this limitation does not compromise relative comparisons among samples processed using the same protocol, future studies should prioritize membrane-based filtration combined with direct microscopic or flow cytometric validation to ensure comprehensive cell recovery.

Finally, taxonomic resolution was restricted to the genus level, as species-level assignments based on partial *16S rRNA* gene sequences are inherently uncertain and were therefore avoided to prevent overinterpretation.

Future investigations integrating absolute microbial quantification, detailed hydrogeochemical measurements, groundwater flow characterization, and functional genomic approaches will be essential to validate and extend the ecological interpretations presented here.

## 5. Conclusions

This study demonstrates that groundwater microbial communities along the Red Sea coastal plain exhibit pronounced vertical and well-specific structuring over short spatial scales, with community composition governed primarily by localized hydrogeochemical and redox conditions rather than spatial distance alone. Across all wells, Proteobacteria (Pseudomonadota) consistently dominated microbial assemblages, with increased representation in deeper strata reflecting selection for metabolically flexible and facultatively anaerobic lineages adapted to suboxic and dynamically fluctuating electron-acceptor regimes.

Wells W1 and W4 displayed higher taxonomic richness, evenness, and broader phylogenetic representation, consistent with relatively stable or transitional redox environments. In contrast, Well W3 exhibited marked community simplification and strong dominance of Gammaproteobacteria and Enterobacteriaceae, indicative of deterministic ecological filtering under localized hydrogeochemical stress or redox instability. These contrasting microbial signatures across a sub-kilometric transect highlight the sensitivity of subsurface ecosystems to fine-scale physicochemical heterogeneity in arid coastal aquifers.

Although archaeal lineages such as Methanobacteriota and Nitrososphaerota occurred at low relative abundance, their detection supports the growing recognition that rare biosphere members are persistent components of groundwater microbiomes and may contribute disproportionately to nitrogen and carbon cycling under suboxic or nutrient-limited conditions. Together, the observed bacterial and archaeal patterns support a conceptual framework in which redox zonation and local hydrogeochemistry act as dominant ecological filters shaping groundwater microbial community structure.

Importantly, microbial patterns identified in this study are based on relative abundance data derived from *16S rRNA* gene amplicon sequencing and should therefore be interpreted as compositional shifts rather than confirmed changes in absolute microbial biomass. Likewise, the absence of direct groundwater flow measurements constrains inference regarding advective transport or hydraulic connectivity among wells. Future studies integrating hydrogeochemical profiling, flow characterization, and absolute microbial quantification (e.g., qPCR or cell-based enumeration), together with functional genomic approaches, will be essential to refine links between microbial community structure, biogeochemical processes, and aquifer vulnerability under increasing environmental and anthropogenic pressures.

## Figures and Tables

**Table 1:** Sampling details and field observations for groundwater wells.

Well	Sampling Level	Depth Below Water Surface	Ambient Air Temperature (°C)	Time	Water Temperature (°C)	Visible Turbidity/Colour	Usage
W1	Surface (S1, S2)	~20 cm	22	07:37	30	Yes	Not used
	Depth (D1, D2)	2.0 m	22	07:52	28	Yes	Not used
W3	Surface (S1, S2)	~20 cm	24	08:46	30	Yes	Not used
	Depth (D1, D2)	1.5 m	24	08:53	28	Yes	Not used
W4	Surface (S1, S2)	~20 cm	24	09:08	30	No	Livestock watering
	Depth (D1, D2)	2.5 m	24	09:16	27	No	Livestock watering

Note: Surface (S1–S2) and depth (D1–D2) denote two independent replicate samples collected at each vertical stratum.

**Table 2:** Alpha diversity metrics of surface and depth groundwater samples from wells W1, W3, and W4.

Well	Sample ID	ASVs	Chao1	Shannon	Faith’s PD	Simpson	Gini–Simpson
W1	W1-S1	480	570.38	5.31	86.78	0.058	0.942
	W1-S2	506	562.03	5.38	89.31	0.060	0.940
	W1-D1	340	362.29	5.03	67.77	0.067	0.933
	W1-D2	450	545.39	4.90	82.22	0.102	0.898
W3	W3-S1	203	237.03	3.48	38.59	0.207	0.793
	W3-S2	181	203.00	3.21	35.98	0.304	0.696
	W3-D1	112	116.09	3.59	20.17	0.154	0.846
	W3-D2	130	131.50	4.30	30.17	0.111	0.889
W4	W4-S1	548	622.78	5.48	88.62	0.056	0.944
	W4-S2	457	506.44	5.11	73.50	0.061	0.939
	W4-D1	402	459.27	4.85	70.02	0.073	0.927
	W4-D2	403	435.25	4.61	67.75	0.119	0.881

## Data Availability

The processed *16S rRNA* gene amplicon sequence data generated in this study have been deposited in the NCBI BioProject database under accession number PRJNA1400952, (http://www.ncbi.nlm.nih.gov/bioproject/1400952, accessed on 1 January 2026) with individual samples registered in the NCBI BioSample database (accessions SAMN54558323–SAMN54558334). The data will be released upon manuscript acceptance.

## Supplementary Materials

The following supporting information can be downloaded at: https://www.mdpi.com/article/10.3390/microorganisms14010232/s1, Figure S1. Field photographs illustrate groundwater well structures and in situ sampling procedures along the Red Sea coastal plain. (A–C) Representative images of Wells W1, W3, and W4 showing well construction and water appearance at the time of sampling. (D) Groundwater sample collection using a weighted container lowered into the well. (E) Depth sampling setup illustrates the rope-mounted container and balloon seal mechanism. (F) In situ measurement of groundwater temperature during sampling. Figure S2. Stacked bar plot showing the relative abundance of bacterial and archaeal classes across individual groundwater samples from wells W1, W3, and W4. Each bar represents a single sample collected from surface (S1, S2) or depth (D1, D2) intervals and analyzed independently (no pooling). Classes with low relative abundance were grouped as “Others” for visualization clarity. Relative abundances were calculated from rarefied *16S rRNA* gene amplicon sequence variant (ASV) tables. Figure S3. Stacked bar plot depicting the relative abundance of dominant bacterial genera across individual groundwater samples from wells W1, W3, and W4. Each bar corresponds to a single surface (S1, S2) or depth (D1, D2) sample analyzed independently. Genera contributing <2% relative abundance per sample were grouped as “Others.” Taxonomic assignments are based on partial *16S rRNA* gene sequences and are reported at the genus level only. Figure S4. Rarefaction curves showing amplicon sequence variant (ASV) richness for individual groundwater samples from wells W1, W3, and W4. Surface (S1–S2) and depth (D1–D2) samples represent biological replicates within each depth interval. Curves illustrate sequencing depth versus observed ASVs for each sample. Replicate samples within the same depth interval display similar rarefaction trajectories, indicating consistent richness estimates. No statistical comparisons were performed. Table S1. Summary of sequencing quality control and read retention across groundwater samples from wells W1, W3, and W4. Table S2. SIMPER analysis identifying genera contributing to Bray–Curtis dissimilarity between groundwater wells.

## Abbreviations

The following abbreviations are used in this manuscript:

QC	Quality control
PCoA	Principal coordinates analysis
UPGMA	Unweighted Pair Group Method with Arithmetic Mean
ASVs	Amplicon sequence variants

## References

[1] Stegen J.C., Fredrickson J.K., Wilkins M.J., Konopka A.E., Nelson W.C., Arntzen E.V., Chrisler W.B., Chu R.K. (2016). Groundwater–surface water mixing shifts ecological assembly processes and stimulates organic carbon turnover. Nat. Commun..

[2] Griebler C., Avramov M. (2015). Groundwater ecosystem services: A review. Freshw. Sci..

[3] Retter A., Karwautz C., Griebler C. (2021). Groundwater microbial communities in times of climate change. Curr. Issues Mol. Biol..

[4] Farnleitner A.H., Wilhartitz I.C., Ryzinska G., Kirschner A.K.T., Stadler H., Burtscher M.M., Hornek R., Szewzyk U., Herndl G.J., Mach R.L. (2005). Bacterial dynamics in spring water of alpine karst aquifers indicates the presence of stable autochthonous microbial endokarst communities. Environ. Microbiol..

[5] Flynn T.M., Sanford R.A., Ryu H., Bethke C.M., Levine A.D., Ashbolt N.J., Santo Domingo J.W. (2013). Functional microbial diversity explains groundwater chemistry in a pristine aquifer. BMC Microbiol..

[6] Esser M.D. (2025). Assessing Microbial Community Dynamics and Functional Shifts Due to Wastewater Discharge Using Advanced Molecular Techniques. Doctoral Dissertation.

[7] Suter E.A., Pachiadaki M., Taylor G.T., Astor Y., Edgcomb V.P. (2018). Free-living chemoautotrophic and particle-attached heterotrophic prokaryotes dominate microbial assemblages along a pelagic redox gradient. Environ. Microbiol..

[8] Herrmann M., Rusznyák A., Akob D.M., Schulze I., Opitz S., Totsche K.U., Küsel K. (2015). Large fractions of CO_2_-fixing microorganisms in pristine limestone aquifers appear to be involved in the oxidation of reduced sulfur and nitrogen compounds. Appl. Environ. Microbiol..

[9] Anantharaman K., Brown C.T., Hug L.A., Sharon I., Castelle C.J., Probst A.J., Thomas B.C., Singh A., Wilkins M.J., Karaoz U. (2016). Thousands of microbial genomes shed light on interconnected biogeochemical processes in an aquifer system. Nat. Commun..

[10] Müller A.L., Kjeldsen K.U., Rattei T., Pester M., Loy A. (2015). Phylogenetic and environmental diversity of *DsrAB*-type dissimilatory (bi)sulfite reductases. ISME J..

[11] Zhou J., Deng Y., Shen L., Wen C., Yan Q., Ning D., Qin Y., Xue K., Wu L., He Z. (2016). Temperature mediates continental-scale diversity of microbes in forest soils. Nat. Commun..

[12] Al-Zahrani K.H., Baig M.B. (2011). Water in the Kingdom of Saudi Arabia: Sustainable management options. J. Anim. Plant Sci..

[13] FAO (2017). Groundwater Governance: A Global Framework for Country Action.

[14] Qian Y., Wang L., Sun J., Gao S., Zhang Y., Liu J. (2022). Microbial community structure and co-occurrence patterns in saline groundwater under different hydrogeochemical conditions. Sci. Total Environ..

[15] Brad T., Griebler C., Hillebrand O., Lueders T. (2008). Microbial community composition and function in groundwater aquifers along a catchment gradient. Environ. Microbiol..

[16] Korbel K.L., Chariton A.A., Stephenson S., Greenfield P., Hose G.C. (2017). Wells provide a distorted view of life in the aquifer: Implications for sampling, monitoring, and assessment of groundwater ecosystems. Sci. Rep..

[17] Yan H., Zhang L., Wu L., Liu Y., Wang S. (2020). Hydrogeochemical controls on microbial community structure in groundwater: Evidence from coastal aquifers of eastern China. J. Hydrol..

[18] Alharbi T., Abdelrahman K., El-Sorogy A.S., Ibrahim E. (2023). Contamination and health risk assessment of groundwater along the Red Sea coast, Northwest Saudi Arabia. Mar. Pollut. Bull..

[19] Al-Hazmi A., Baig M.I., Al-Ghamdi A. (2021). Hydrochemical characterization and groundwater quality assessment for sustainable use in arid coastal aquifers of the Red Sea region. Groundw. Sustain. Dev..

[20] Zhang H., Lv Y., Zhang T., Zhang L., Ma X., Liu X., Lian S. (2024). Characteristics of Groundwater Microbial Community Composition and Environmental Response in the Yimuquan Aquifer, North China Plain. Water.

[21] Schroer H.W., Markland K., Ling F., Just C.L. (2025). Hydraulic Connectivity and Hydrochemistry Influence Microbial Community Structure in Agriculturally Affected Alluvial Aquifers in the Midwestern United States. Environ. Sci. Technol..

[22] Wang H., Herrmann K., Ivanova A., Schroeter C., Zabel C., Lehmann R., Kästner M., Horner G., Gleixner G., Totsche K.U. (2025). Groundwater microbiomes balance resilience and vulnerability to hydroclimatic extremes. Commun. Earth Environ..

[23] Wang Y., Wang Y., Shang J., Wang L., Li Y., Wang Z., Zou Y., Cai W., Wang L. (2024). Redox gradients drive microbial community assembly patterns and molecular ecological networks in the hyporheic zone of effluent-dominated rivers. Water Res..

[24] Zhao Y., Li D. (2025). Seawater intrusion regulates microbial community structure and functional potential in subterranean estuaries of the Yangtze River. Front. Microbiol..

[25] Zhi C., Hu X., Yang F., Huang X., Chen H., Chen L., Chen G., Wu Z., Wang S. (2024). Unraveling microbial community variation along a salinity gradient and indicative significance to groundwater salinization in the coastal aquifer. J. Hydrol..

[26] Fan W., Yan S., Gao B., Xiu W., Zhao Y., Guo H. (2023). Linking groundwater microbiome and functional ecological clusters to geogenic high hexavalent chromium from deep aquifers in a loess plateau. Water Res..

[27] Stegen J.C., Lin X., Konopka A.E., Fredrickson J.K. (2012). Stochastic and deterministic assembly processes in subsurface microbial communities. ISME J..

[28] Zhang L.Z., Xing S.P., Huang F.Y., Xiu W., Lloyd J.R., Rensing C., Zhao Y., Guo H. (2024). Hydrogeochemical differences drive distinct microbial community assembly and arsenic biotransformation in unconfined and confined groundwater of the geothermal system. Sci. Total Environ..

[29] Clooney A.G., Fouhy F., Sleator R.D., O’Driscoll A., Stanton C., Cotter P.D., Claesson M.J. (2016). Comparing apples and oranges? Next generation sequencing and its impact on microbiome analysis. PLoS ONE.

[30] Song Q., Zhou B., Song Y., Du X., Chen H., Zuo R., Zheng J., Yang T., Sang Y. (2025). Microbial community dynamics and bioremediation strategies for petroleum contamination in an in-service oil depot, middle–lower Yellow River Basin. Front. Microbiol..

[31] de Gouveia M.I.M. (2022). Exploring the Metabolic Mechanisms Used by Enterobacteriaceae to Thrive in the Dysbiotic Gut of Patients Suffering from Intestinal or Extra-Intestinal Inflammatory Diseases. Doctoral Dissertation.

[32] Baker B.J., De Anda V., Seitz K.W., Dombrowski N., Santoro A.E., Lloyd K.G. (2020). Diversity, ecology and evolution of Archaea. Nat. Microbiol..

[33] Spiridonov V., Ćurić M., Novkovski N. (2025). Biosphere: Ecosystem Diversity and Environmental Change. Atmospheric Perspectives: Unveiling Earth’s Environmental Challenges.

[34] Mosley O.E., Gios E., Weaver L., Close M., Daughney C., van der Raaij R., Martindale H., Handley K.M. (2022). Metabolic diversity and aero-tolerance in anammox bacteria from geochemically distinct aquifers. mSystems.

