# Supplementary material for: Spatial and Vertical Stratification of Groundwater Microbial Communities Reveals Proteobacterial Dominance and Redox-Driven Ecological Transitions
Source: Microorganisms. 2026 Jan 19;14(1):232. doi: 10.3390/microorganisms14010232 (PMC12843820; doi:10.3390/microorganisms14010232)
Supplement: Supplementary file 1 [file microorganisms-14-00232-s001.zip › microorganisms-4080623-supplementary.pdf]

Table S1. Summary of sequencing quality control and read retention across groundwater samples from wells W1, W3, and W4.

Sample Name	Raw data	Adapter & Primer Trimming	Preprocessing Length Trimming	Quality Filter	QC Remain	Denoised For	Denoised Rev	Merged Pair	Non-chimeric	ASV Length Filter	ASV Remain
W1-S1	195,601	193,423	193,423	164,516	84.11%	159,331	161,657	148,902	106,154	106,154	54.27%
W1-S2	143,333	141,775	141,775	122,240	85.28%	117,657	119,817	108,488	76,658	76,658	53.48%
W1-D1	128,753	127,560	127,560	91,857	71.34%	90,201	91,115	87,084	75,226	75,226	58.43%
W1D2	169,658	168,076	168,076	145,313	85.65%	144,393	144,607	143,317	141,485	141,485	83.39%
W3-S1	157,925	156,111	156,111	135,064	85.52%	133,711	134,249	129,521	97,184	97,184	61.54%
W3-S2	141,110	139,550	139,550	122,884	87.08%	121,673	122,096	115,735	75,354	75,350	53.40%
W3-D1	119,477	118,123	118,123	84,550	70.77%	84,066	84,216	82,066	65,264	65,264	54.62%
W3D2	117,110	116,113	116,113	88,651	75.70%	88,588	88,610	88,181	88,181	88,181	75.30%
W4-S1	157,887	156,255	156,255	136,708	86.59%	133,145	134,918	123,016	95,969	95,969	60.78%
W4-S2	166,811	165,005	165,005	142,235	85.27%	139,820	140,967	132,632	107,273	107,271	64.31%
W4D1	151,004	149,206	149,206	129,064	85.47%	126,492	127,769	119,285	93,498	93,498	61.92%
W4D2	155,710	154,068	154,068	132,713	85.23%	131,459	131,831	127,932	117,133	117,133	75.23%

Note: Chloroplast- and mitochondria-affiliated sequences were removed during taxonomic filtering prior to rarefaction. Organelle-derived reads were absent from the final dataset (0.00% across all samples), indicating negligible host or phototrophic contamination.

Table S2. SIMPER analysis identifying genera contributing to Bray–Curtis dissimilarity between groundwater wells

Comparison	Genus	Mean abundance (W1)	Mean abundance (W3)	Average contribution (%)	Cumulative contribution(%)
**W1 vs. W3**	Rheinheimera	0.06	0.33	18.4	18.4
	Escherichia	0.01	0.36	16.2	34.6
	Polynucleobacter	0.07	0.01	12.1	46.7
	Cellulophaga	0.03	0.09	9.6	56.3
	Limnohabitans	0.07	0.01	8.1	64.4
	Actinomycetota_g_Other	0.16	0.09	6.5	70.9
	Comamonadaceae_g_Other	0.06	0.03	5.4	76.3
	Aestuariivirga	0.04	0.01	4.1	80.4

Note: SIMPER analysis was performed based on Bray–Curtis dissimilarity using genus-level relative abundance data. Mean abundances represent average relative abundances within each well. Average contribution (%) indicates the mean contribution of each genus to the overall dissimilarity between wells, while cumulative contribution (%) represents the running total of explained dissimilarity. Only taxa cumulatively contributing up to approximately 80% of the total dissimilarity are shown.

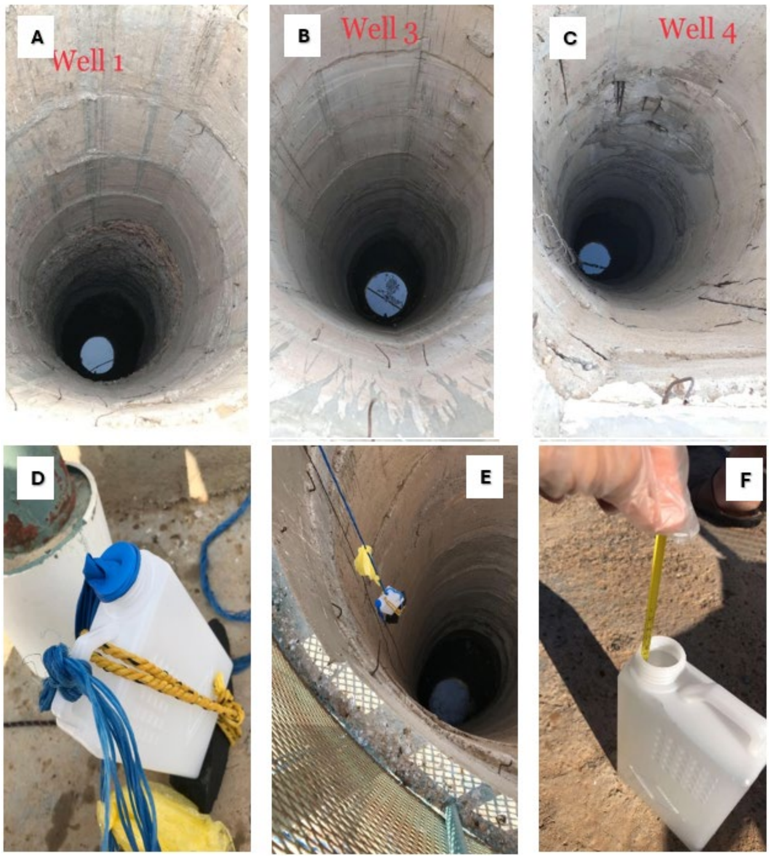

Figure S1. Field photographs illustrate groundwater well structures and in situ sampling procedures along the Red Sea coastal plain. (A–C) Representative images of Wells W1, W3, and W4 showing well construction and water appearance at the time of sampling. (D) Groundwater sample collection using a weighted container lowered into the well. (E) Depth sampling setup illustrates the rope-mounted container and balloon seal mechanism. (F) In situ measurement of groundwater temperature during sampling.

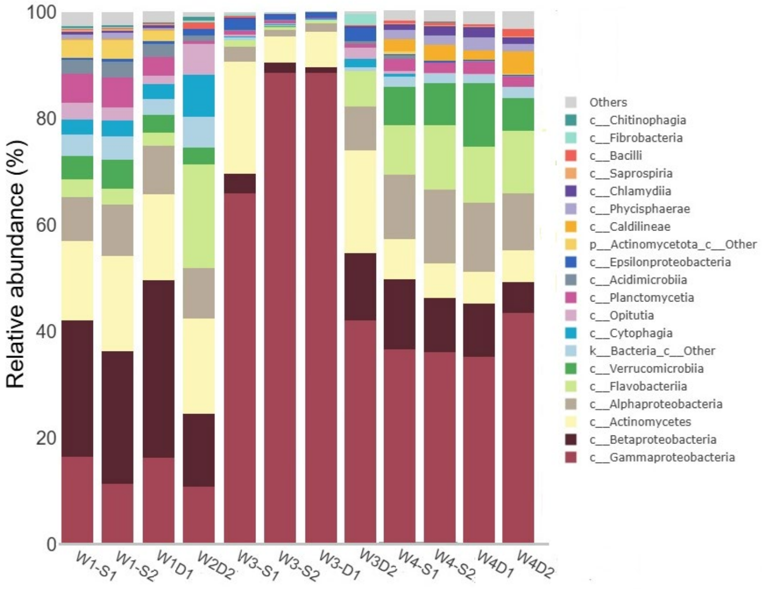

<u>Figure S2.</u> Stacked bar plot showing the relative abundance of bacterial and archaeal classes across individual groundwater samples from wells W1, W3, and W4. Each bar represents a single sample collected from surface (S1, S2) or depth (D1, D2) intervals and analyzed independently (no pooling). Classes with low relative abundance were grouped as "Others" for visualization clarity. Relative abundances were calculated from rarefied 16S rRNA gene amplicon sequence variant (ASV) tables.

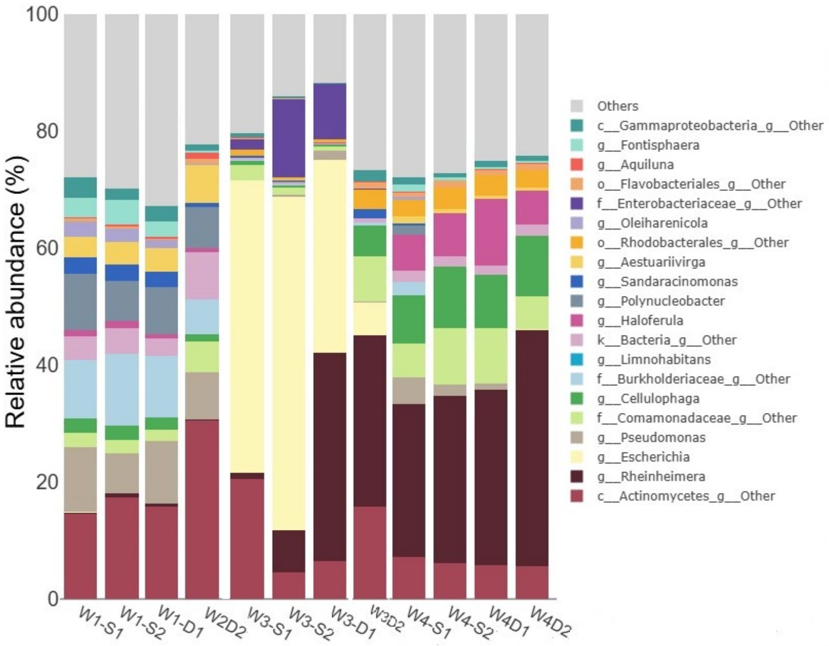

<u>Figure S3</u>. Stacked bar plot depicting the relative abundance of dominant bacterial genera across individual groundwater samples from wells W1, W3, and W4. Each bar corresponds to a single surface (S1, S2) or depth (D1, D2) sample analyzed independently. Genera contributing <2% relative abundance per sample were grouped as "Others." Taxonomic assignments are based on partial 16S rRNA gene sequences and are reported at the genus level only.

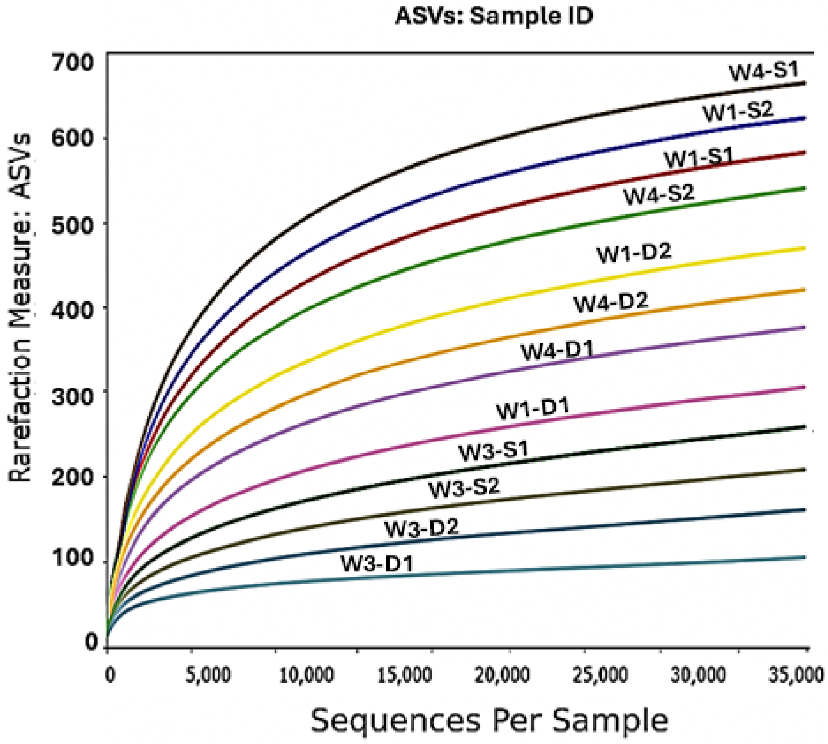

Figure S4. Rarefaction curves showing amplicon sequence variant (ASV) richness for individual groundwater samples from wells W1, W3, and W4. Surface (S1–S2) and depth (D1–D2) samples represent biological replicates within each depth interval. Curves illustrate sequencing depth versus observed ASVs for each sample. Replicate samples within the same depth interval display similar rarefaction trajectories, indicating consistent richness estimates. No statistical comparisons were performed.
